# Trigger Points: An Anatomical Substratum

**DOI:** 10.1155/2015/623287

**Published:** 2015-02-24

**Authors:** Flávia Emi Akamatsu, Bernardo Rodrigues Ayres, Samir Omar Saleh, Flávio Hojaij, Mauro Andrade, Wu Tu Hsing, Alfredo Luiz Jacomo

**Affiliations:** ^1^Department of Surgery, Laboratory of Medical Research, Division of Human Structural Topography, Faculty of Medicine of the University of São Paulo (FMUSP), 04717-003 São Paulo, SP, Brazil; ^2^Postgraduate Program of the Surgical Clinic of FMUSP, 01246-903 São Paulo, SP, Brazil; ^3^Department of Surgery Medicine, Laboratory of Medical Research, FMUSP, 01246-903 São Paulo, SP, Brazil; ^4^Department of Pathology, Discipline of Telemedicine, FMUSP, 01246-903 São Paulo, SP, Brazil

## Abstract

This study aimed to bring the trapezius muscle knowledge of the locations where the accessory nerve branches enter the muscle belly to reach the motor endplates and find myofascial trigger points (MTrPs). Although anatomoclinical correlations represent a major feature of MTrP, no previous reports describing the distribution of the accessory nerve branches and their anatomical relationship with MTrP are found in the literature. Both trapezius muscles from twelve adult cadavers were carefully dissected by the authors (anatomy professors and medical graduate students) to observe the exact point where the branches of the spinal accessory nerve entered the muscle belly. Dissection was performed through stratigraphic layers to preserve the motor innervation of the trapezius muscle, which is located deep in the muscle. Seven points are described, four of which are motor points: in all cases, these locations corresponded to clinically described MTrPs. The four points were common in these twelve cadavers. This type of clinical correlation between spinal accessory nerve branching and MTrP is useful to achieve a better understanding of the anatomical correlation of MTrP and the physiopathology of these disorders and may provide a scientific basis for their treatment, rendering useful additional information to therapists to achieve better diagnoses and improve therapeutic approaches.

## 1. Introduction

Myofascial pain syndrome (MPS) is the most frequent cause of chronic musculoskeletal pain [1–6], with estimates of world-wide prevalence ranging from 0.5% to 5.0% [6–8]. MPS is defined as a focal hyperirritability in muscle tissue [9]. This syndrome presents clinically as referred pain [7], a limited range of motion in joints [10], and a local twitch response following mechanical stimulation of certain muscular and fascial areas, known as myofascial trigger points (MTrPs) [5], and is associated with motor endplates [11]. The chronic pain diagnostic taxonomy published by the International Association for the Study of Pain provides no specific criteria for the diagnosis of MPS; rather, it offers a note that the diagnosis depends upon the demonstration of a trigger point and reproduction of pain by maneuvers which place stress upon proximal structures or nerve roots [12]. The lack of a formal, widely accepted, criterion-based diagnostic scheme has proved to be a serious impediment to proper diagnosis, clinical communication, and research related to this topic [13].

Four different MTrPs related to motor innervation have been clinically described for the trapezius muscle [13], but data showing anatomical correlations are still lacking [14, 15]. The complex pathophysiology of MTrPs is not yet fully understood, but it is theoretically based on the presence and activity of MTrP [3, 16].

Clinically, MTrPs affecting the trapezius muscle may cause pain in the neck, shoulders, proximal portion of the arms, dorsal region, and headache (generally considered to be tension type or cervicogenic), as well as temporomandibular disorder, limitation of the movement of the cervical segment, specially rotation, and dizziness [12, 13].

MTrPs are clinically identified by palpation of a knot within a taut band of muscle or fascia that elicits local tenderness and referred pain [3, 5, 17, 18], and this criterion is often used in studies that try to assess the prevalence of the MTrPs. One such study tried to determine the role of the MPS in head, neck, and upper extremities symptoms presented by workers, both blue-collar and white-collar, and found that active MTrPs of the trapezius muscle were the most common and could be responsible for their pain [19]. This is consistent with the description given by Travell and Simons that this muscle is one of the most affected (if not the single most affected) by the condition [13].

Electrophysiological analyses have been published suggesting that an MTrP occurs when a nociceptor and a muscular motor endplate coincide [11, 18, 20–22]. Nevertheless, the lack of anatomical information about these structures constitutes a major obstacle to obtaining a complete understanding of the pathophysiology and widespread clinical treatment of MTrP.

Our hypothesis was that MTrPs could be related to muscle innervation. The present study's goal is to provide the anatomical basis of the MTrP, supplying data that could be used in future studies in order to understand the syndrome, its mechanisms, and, ultimately, how to better treat it.

## 2. Methods

Twelve human adult cadavers (6 males and 6 females) were dissected to expose the dorsal primary rami that innervate the trapezius muscle after branching off the spinal nerve. The cadavers were previously prepared using a 4% phenolic acid and 0.5% formaldehyde solution. The cadavers were obtained from a body donation program undertaken by the Discipline of Human Structural Topography of the Department of Surgery of the University of São Paulo Medical School, and this study was approved by the Ethics Committee on Research of the University of São Paulo Medical School under protocol 130/11. Specimens with no sign of previous surgery or any other severe abnormality in the regions of interest were accepted. The specimens were positioned in ventral decubitus on the dissection table, and a long incision down the spine was made starting from the external occipital protuberance and continuing to the T12 level. Next, flaps of skin and subcutaneous tissue were pulled away laterally to expose the trapezius muscles from both sides. We cut through the attachment of the trapezius to the spines of the vertebrae and then folded the muscle laterally, without damaging the underlying neurovasculature. The spinal accessory nerve arises in the neck through the jugular foramen, descends along the cervical region under the sternocleidomastoid muscle, and reflects to the posterior triangle of the neck in the distal third of that muscle, reaching the shoulder where it extends its first branch to the trapezius muscle, which we designated MTrP1. This denomination is also employed in clinical settings and corresponds to the anatomical location described in the specimens dissected in this study. Photographic documentation was obtained from these nervous structures. Dissections to identify the exact point where fibers from the spinal accessory nerve enter the trapezius muscle were extremely difficult because of the intricate relationship among the nerve fibers, fascia, and fat. The dissections usually required 3 to 4 periods of 4 hours of careful work under magnifying lenses to thoroughly study the specimens. We kept the numbers of each MTrP as it was described by Travell, for better comparison.

## 3. Results

We observed in all of the cadavers that the nerve fibers reached the muscle and tendon in areas coincident with the clinical locations of MTrPs described by Simons [5, 11, 18, 20].

**Figure 1:**
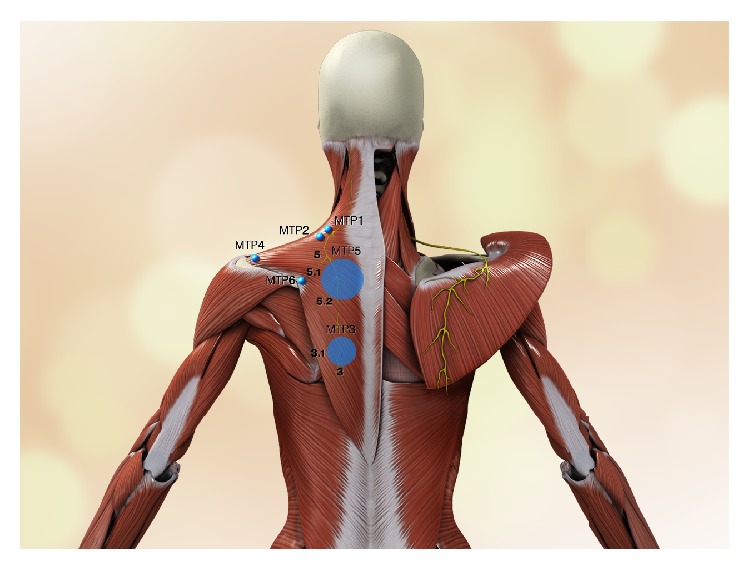
Scheme of anatomical location of MTrP1, MTrP2, MTrP3, MTrP3.1, MTrP4, MTrP5, MTrP5.1, MTrP5.2, and MTrP6. Motor endplate locations correspond to MTrP1, MTrP2, MTrP3, MTrP3.1, MTrP5, MTrP5.1, and MTrP5.2. MTrP4 and MTrP6 are insertional trigger points. Observe on the left side that points 5 and 3 are regions that correspond to nerve insertional points. The accessory nerve and its branches are highlighted in yellow.

**Figure 2:**
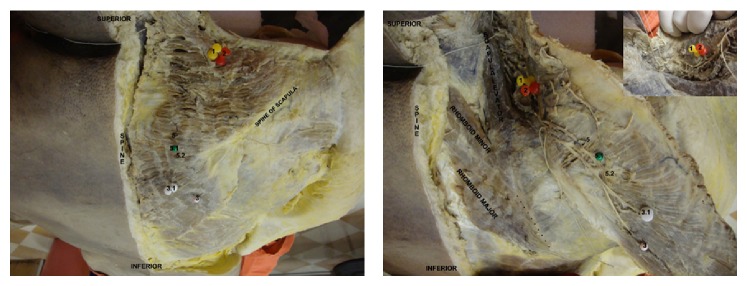
Gross anatomy image corresponding to the previous scheme. Dissection of the trapezius muscle and the areas corresponding to the clinical trigger points. Reflection of the right trapezius muscle showing the entry points of the spinal accessory nerve branches that coincide with the MTrPs 1, 2, 3, 3.1, 5, 5.1, and 5.2. Trapezius muscle, levator scapulae, rhomboid major, and rhomboid minor are identified.

All four MTrPs located in the muscle belly in all cadavers and both insertional MTrPs were identified as entry points of the spinal accessory nerve into the trapezius muscle and tendon. The locations of the entry points coincided with the clinical area of MTrPs. Variation was observed in two MTrPs: point 3 presented two nervous entry points, designated MTrP3 and MTrP3.1, and point 5 presented three different patterns, designated MTrP5, MTrP5.1, and MTrP5.2. MTrP1 was situated in the intermediate part of the anterior border of the upper part of the muscle, as described previously (Figures 1 and 2). MTrP2 was located in a distal and slightly lateral position to the previous MTrP (Figures 1 and 2). MTrP3 corresponded to entry points on the border of medial fibers near the inferior border of the muscle, and two nerve branches were found in this region in all specimens (Figures 1 and 2). Similarly, MTrP5, located in the intermediate part of the muscle belly, presented three different nerve entries (Figures 1 and 2). The insertional MTrP4 and 6 corresponded to the muscle insertions in the spine of the scapula and acromion (Figure 2). This was a constant pattern observed in our dissection for all cadavers (Table 1 and Figure 1).

## 4. Discussion

Myofascial trigger points are considered to represent a pathogenic model of pain from diverse etiologies [23]. MTrPs, or muscle knots, are a type of electrically active, hyperirritable areas of muscle associated with contractile nodules and dysfunctional motor endplates [5]. Theoretically, a sensitive spot may be found anywhere in the skeletal muscle but is usually found near the motor endplates [3, 5, 17, 18, 24]. MTrP can be either active, causing painful disorders, or latent, causing pain only when stimulated [2, 5, 7]. Clinical identification is the most commonly used technique to locate MTrP, and both needle and surface electromyography have been described for research purposes [4, 15, 17]. Knowledge of the anatomical location of these structures is essential not only to provide a correct diagnosis but also for the sake of many therapeutic modalities including a direct approach of the affected area [1, 3, 5, 6, 9, 17, 21, 25, 26]. Several painful cervical and thoracic disorders are related to trigger points in the trapezius muscle, and misdiagnosis of these conditions is common when the putative responsible MTrPs are not adequately evaluated [6].

The trapezius muscle covers the posterior aspect of the neck and the superior part of the thorax and attaches the upper limb to the skull and vertebrae. Its fibers are divided according to their orientation into superior, intermediate, and inferior fibers, each of which exhibits a different action. The superior fibers have their origin in the superior nuchal line and external occipital protuberance and reach the lateral portion of the clavicle. The intermediate fibers retract the scapula and originate from the spinous processes from the 7th cervical to the 3rd thoracic vertebrae and are inserted on the acromion. These fibers represent the strongest portion of the muscle. The last portion, represented by the inferior fibers, depresses the scapula and lowers the shoulder. Its fibers have their origin in most thoracic spinous processes and are inserted along the spine of the scapula [27, 28]. Seven MTrPs are related to the trapezius muscle [5]. Four points are located in the muscle belly of the trapezius: (1) the mid-portion of the superior margin, extending into the vertical fibers that reach the clavicle; (2) found caudally and laterally to the location of the first point, in the transverse fibers of the muscle; (3) the medial fibers near the inferior margin of the muscle; and (4) the central part of the muscle belly between the C7-T3 levels. Two additional MTrPs are found on the tendinous insertion: (1) over the medial part of the spine on the scapula and (2) on the acromial insertion of the trapezius muscle. There is another MTrP superficially located on the posterior aspect of the middle part of the clavicle [9].

According to our findings, which coincides with what was published by Simons et al. (2005) [5], MTrP1 of the trapezius muscle is found over the medial anterior border of the muscle in its superior portion and concerns the most vertical fibers attached to the clavicle. MTrP2 is caudal and slightly lateral to MTrP1. MTrP3 and MTrP5 were easier to identify in our cadavers; MTrP3 was located near the inferior border of the muscle in its inferior fibers of the muscle, showing two insertional points (Figures 1 and 2) and MTrP5, as expected from its clinical pattern, was found in several different areas, but with three insertional points (Figures 1 and 2) rather than only one insertional point as observed clinically, all belonging to the intermediate portion of the muscle. Despite the variations observed in points 3 and 5, it is possible that the clinical localization of the MTrP to a single area is due to the anatomical proximity of these different nervous entry points, thus resulting in somewhat overlapping electromyographic identification. Therefore, if there is one MTrP3 and one MTrP5 that are clinically relevant, we can state that these MTrPs correspond to two and three motor plates, respectively. This anatomical study was, in accordance with our hypothesis, based on the dissection of adult cadavers to observe where branches of the spinal accessory nerve entered the muscle belly and whether there was a positive correlation with MTrP.

The trapezius muscle has two additional MTrPs corresponding to its tendinous insertion and they can also be responsible for painful disorders, although there is no relation to the end motor plates. MTrP4 and MTrP6 were also observed in our studies, and no anatomical variation was detected. MTrP4 was found on the medial aspect of the spine of the scapula and MTrP6 on the acromion, both of which are sites of tendinous insertion of the trapezius muscle (Figure 1).

The findings from this study reveal that the locations where the accessory nerve enters the belly and tendons of the trapezius muscle are the same locations of the MTrP that can be identified in this muscle. This might have a strong relation to the pathophysiology of the MPS and could also be useful when treating the condition.

We believe that knowledge of the anatomical basis of MTrP is a cornerstone that will help provide a precise map for clinical applications related to certain painful disorders and that further investigation of trigger points in other muscles is needed.

## Figures and Tables

**Table 1:** Anatomical location of the MTrPs in 12 cadavers (insertional MTrPs were not listed).

Trigger point	Specimens 1, 2, 3, 4, 5, 6, 7, 8, 9, 10, 11, and 12/left and right side
MTrP1	Intermediate part of the anterior border of the upper part of the muscle near the vertical fibers of the muscle attached to clavicle
MTrP2	Distal and slightly lateral to MTrP1
MTrP3	Medial to the scapula and on the border of the medial fibers near the inferior border of the muscle
MTrP3.1	Medial fibers near the inferior margin of the muscle, medial to MTrP3; nervous branch always smaller than MTrP3 branch.
MTrP5	Medial to the scapula in the intermediate part of the muscle belly
MTrP5.1	Medial to the scapula in the intermediate part of the muscle belly inferior to MTrP5
MTrP5.2	Medial to the scapula in the intermediate part of the muscle belly inferior to MTrP5.1
